# New device for assessing the toe-brachial index and a pilot cross-sectional study in diabetic patients

**DOI:** 10.1590/1677-5449.202401042

**Published:** 2025-07-28

**Authors:** Thiago Paes de Barros De Luccia, Alexandre Donisete Bensi, Nelson De Luccia, Priscilla Matos Cunha, Lia Maristela da Silva Jacob, Leonardo Servato Sanches Martins de Barros, Julio Maganha Gouvêa, Vívian Mei Matuoka

**Affiliations:** 1 Faculdade São Leopoldo Mandic Araras – FSLM/Araras, Araras, SP, Brasil.; 2 Centro Universitário Central Paulista, São Carlos, SP, Brasil.; 3 Universidade de São Paulo – USP, São Paulo, SP, Brasil.

**Keywords:** toe brachial index, ankle brachial index, arterial pressure, photoplethysmography, índice hálux-braquial, índice tornozelo-braquial, pressão arterial, fotopletismografia

## Abstract

**Background:**

Blood pressure measurements in the hallux and fingers are important in evaluation of patients with diabetes, obese patients, dialysis patients, and those with peripheral artery disease.

**Objectives:**

In this article, we tested a prototype of an automatic device for measuring systolic pressure in fingers and toes, combining optical plethysmography and a pressure sensor, with a cuff encircling the finger or toe, and controlled by a microcontroller.

**Methods:**

The prototypes (a total of 3 identical devices) were tested for initial validation (with a group of participants who already had symptoms of peripheral arterial disease [PAD], n=30) and also in clinical comparisons between controls (n=15) and participants with diabetes (n=15). The following variables were tested: Ankle-brachial index; Toe-finger index; Toe-brachial index, and toe pressure in isolation. For validation, the toe pressure in isolation was measured with two devices (the prototype under test and the SysToe® device), to analyze the correlation between the two measurements.

**Results:**

The correlation between the two devices was strong (R=0.88) in the group with symptoms of PAD. Toe-brachial index was lower in patients with diabetes than in control participants (p=0.005) as was the Toe-finger index (p=0.03), however, the Ankle-brachial index was similar in these two groups (p=0.92).

**Conclusions:**

The findings show the feasibility of the device for measuring finger and toe pressures, and possibly indicate greater sensitivity for detecting early-stage arterial disease using this type of measurement.

## Resumo

**Contexto:**

As medidas de pressão arterial do hálux e dos dedos das mãos são importantes na avaliação de pacientes com diabetes, obesos, em diálise e com doença arterial periférica (DAP).

**Objetivos:**

Neste artigo, testamos um protótipo de dispositivo automático para medição da pressão sistólica nos dedos das mãos e dos pés, associando pletismografia óptica e sensor de pressão, com um manguito envolvendo o dedo da mão ou do pé, controlado por um microcontrolador.

**Métodos:**

Os protótipos (total de três dispositivos idênticos) foram testados quanto à validação inicial (grupo de participantes já com sintomas de DAP, n=30) e também em uma comparação clínica entre controles (n=15) e participantes diabéticos (n=15). As seguintes variáveis foram testadas: índice tornozelo-braquial, índice hálux-digital, índice hálux-braquial e pressão isolada do hálux. Para a validação, a pressão isolada do hálux foi medida com dois dispositivos (protótipo em teste e dispositivo SysToe®), a fim de estabelecer uma correlação entre as duas medidas.

**Resultados:**

A correlação entre os dois dispositivos foi forte (R=0,88) no grupo com sintomas de DAP. O índice hálux-braquial foi menor em diabéticos do que nos controles (p=0,005), assim como o índice hálux-digital (p=0,03). No entanto, o índice tornozelo-braquial foi semelhante entre os dois grupos (p=0,92).

**Conclusões:**

Os achados demonstram a viabilidade do dispositivo para medir as pressões dos dedos das mãos e dos pés, além de indicar uma possível maior sensibilidade para detectar doença arterial em estágio inicial com esse tipo de medição.

## INTRODUCTION

The prevalence of peripheral arterial disease (PAD) has been increasing worldwide over recent years.^1^ In a national, multicenter, cross-sectional study with 1,170 individuals (>18 years) in Brazil, the prevalence of PAD was 10.5%. In this study, PAD was diagnosed using the ankle-brachial index (ABI) < or = 0.90.^2^

Blood pressure measurements in the hallux and fingers are important in the evaluation of patients with diabetes, obese patients, dialysis patients, and those with peripheral artery disease. The toe-brachial index is useful when the more commonly used ankle-brachial index is abnormally elevated (>1.3) due to non-compressible arteries.^3^ Measurement of hallux pressure in isolation is also important for assessing the prognosis of wound healing in patients with diabetes.^4^ Finger blood pressure measurements are also clinically useful for assessing upper limb arterial status in patients with systemic sclerosis and with arteriovenous fistulas in arms.^5,6^ Screening for peripheral arterial occlusive disease in patients with diabetes using the toe-brachial index is still a subject under study.^7^ The toe-brachial index has been considered superior to the ankle-brachial index measurement for this type of screening.^8^

In this article, we tested a prototype of an automatic device for measuring systolic pressure in fingers and toes, developed by some of the study authors. There are some devices that measure pressure in the hallux using photoplethysmography and laser Doppler sensors. It appears that only one device on the market was developed exclusively for automatically measuring hallux blood pressure, the SysToe (Atys Medical, France). This device has already been validated in research^9,10^ and will be our standard for comparison.

## METHODS

**Figure 1:**
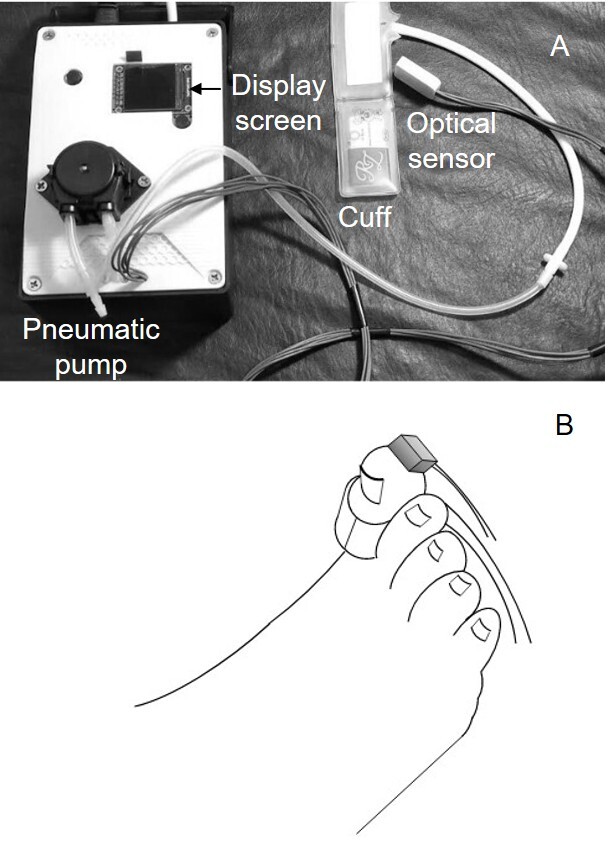
(A) Prototype under test; (B) Plethysmography sensor and cuff around the hallux.

**Figure 2:**
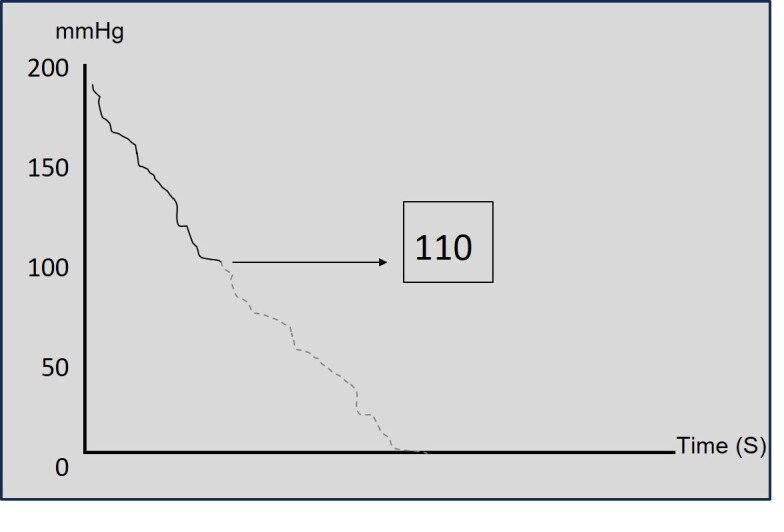
Representation of the trace on a Cartesian graph. At the point that blood flow is detected, the trace changes color (from solid to dashed line in the image) and the numerical blood pressure measurement appears on the screen (here we show an example systolic pressure in the hallux of 110 mmHg). Abscissa: time (in seconds); ordinate: pressure in millimeters of mercury (mmHg).

The prototype uses the MAX30102EFD+ optical plethysmography sensors (Maxim Integrated, United States) associated with the MPRLS0025PA00001 pressure sensor (Honeywell, United States) and the ATMEGA328P microcontroller (Microchip Technology, United States). The cuff that surrounds the finger is inflated and deflated by a pneumatic electric pump, controlled by the microcontroller. The inflatable cuffs can be changed to match finger/toe diameters (Figures 1A and 1B). The goal is for the inflatable part of the cuff to cover 80 to 100% of the circumference of the finger or toe. The pneumatic pump initially inflates the cuff to 200 mmHg, and then begins to gradually deflate (this is when the pressure decay graph is generated). The display screen shows the pressure trace on a Cartesian graph (abscissa: time (in seconds); ordinate: pressure in millimeters of mercury [mmHg]). When blood flow is detected by the photoplethysmography sensor, the trace changes color and the numerical blood pressure measurement appears on the screen (In Figure 2, the line changes from solid to dashed to represent this color change).

**Figure 3:**
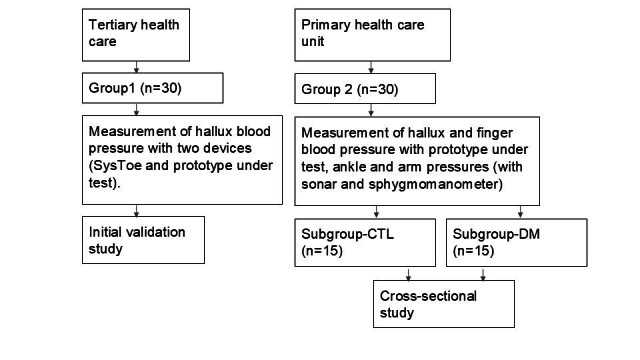
Flowchart illustrating how the studies were carried out.

Tests with the prototypes (total of 3 identical prototypes) were conducted at two medical centers, a high complexity hospital - Hospital das Clínicas, Faculty of Medicine, University of São Paulo (São Paulo/SP) (tertiary health care) and a community health center in Araras, SP, (PSF Dr. Edmundo Ulson, Araras/SP) (primary health care). In the present study, we sought to carry out initial validation of a medical device prototype (in tertiary health care, group 1) and initial clinical research (in primary health care, group 2). Figure 3 shows a flowchart illustrating how the studies were carried out.

In the tests in tertiary health care (initial validation), a single group (group 1) was formed of patients who already had symptoms of PAD (n=30), with which an initial prototype validation study was conducted (a prospective validation study). Patients were examined by the vascular surgery team and pain, weakness, tingling, or ulceration were considered symptoms of peripheral arterial disease. The ESH-IP document^11^ establishes a sample size for the validation of blood pressure measuring devices of approximately 30 individuals, with multiple blood pressure measurements performed on the same individual. The measurements recorded for the prototype under test were each the arithmetic means of two to three measurements taken from the same participant. Inclusion criteria were adult men and women and non-pregnant women. In this group we evaluated toe arterial pressure only using two devices (the prototype under test and the SysToe device) to build a correlation between the two device’s measurements. Pearson’s correlation coefficient was used to calculate test reliability. Additionally, initial measures of accuracy (sensitivity, specificity, and positive predictive value) were calculated in accordance with STARD document recommendations.^12^ To create a 2x2 diagnostic accuracy table, a “sick” person was defined as one who had a toe pressure lower than 50mmHg, measured with the standard device (SysToe). For both devices (SysToe and prototype), values measured close to the cut-off point (50 mmHg) were discarded if discrepant by less than 10 mmHg. During data collection for the initial prototype validation, toe blood pressure only was measured in patients with symptoms of peripheral arterial disease for reasons of convenience and logistics. This group of patients was essential for the initial validation and to construct correlations, since it enabled measurement of toe blood pressures ranging from low (<50 mmHg) to those considered normal (70 to 110 mmHg).

For the tests conducted at the primary health care unit (group 2), two distinct subgroups were formed (participants without diabetes-Controls [CTL, n=15] and patients with diabetes [DM, n=15]). A comparative cross-sectional study of clinical variables was conducted to compare these subgroups. Data were collected from each individual at a single visit to the community health center from May 2023 to April 2024. The required sample size was calculated as 48 controls and 48 diabetes patients, but as this is a pilot study, we adopted a convenience sample of 15 CTL and 15 DM. An extension of this clinical study is already underway with the calculated sample sizes. In these two subgroups, we evaluated the variables ankle-brachial index, toe-brachial index, and toe-finger index with the prototype under test only. Inclusion criteria were adult men and women, non-pregnant women, with or without diabetes. The *t* test for independent samples was used to compare the CTL and DM subgroups.

The study was approved by the ethics committee at the Faculty São Leopoldo Mandic-Araras/SP, CAAE 68976723.1.0000.5374 (report number: 7303065), and by the ethics committee at the Faculty of Medicine, University of São Paulo (cep/conep 05-01-2022). All participants signed an informed consent form. The toe pressure measurement device (prototype under test) was developed with funding from the PIPE-FAPESP program (Process Number: 2020/05856-4).

## RESULTS

**Figure 4:**
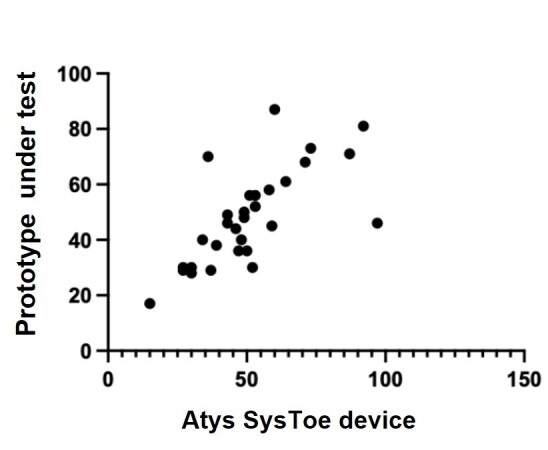
Correlation between the two devices (prototype under test and SysToe device). Pearson’s correlation coefficient, R=0.88. Group with symptoms of PAD (group 1).

Regarding the comparison between the two devices, the correlation showed good agreement between hallux blood pressure measurements in group 1 (with symptoms of PAD). Pearson’s correlation coefficient was R=0.88. In Figure 4, we can observe about 3 points that are distant from the well-formed correlation cloud. Regarding the accuracy measures for this small initial sample, sensitivity was 0.78, specificity was 0.83, and the positive predictive value was 0.83. To construct the 2 x 2 table of diagnostic accuracy, the following data were considered: “sick” participants (toe pressure <50mmHg) = 15; false negatives = 4; healthy participants (toe pressure >50mmHg) = 15; false positive = 3. Table 1 shows the raw data used for the correlation analysis.

**Table 1:** Data used for correlation analysis in group 1. Toe blood pressure measurements (mmHg) taken with the standard device (SysToe) and the prototype under test are presented.

SysToe	Prototype under test
15.1	15.7
27.3	27.1
30.3	25.7
25.7	28.5
30.3	28.6
37.9	30.0
39.4	37.5
33.3	42.0
53.0	30.0
46.9	36.0
50.0	37.5
48.5	40.5
45.4	46.5
43.9	58.5
43.9	57.0
50.0	48.0
48.5	51.0
54.5	52.5
50.0	55.5
54.5	58.5
65.1	60.0
63.6	61.5
36.4	70.5
71.2	67.5
74.2	73.5
60.6	88.5
98.5	46.5
87;9	72.0
92.4	81.0
93.9	81.0

**Figure 5:**
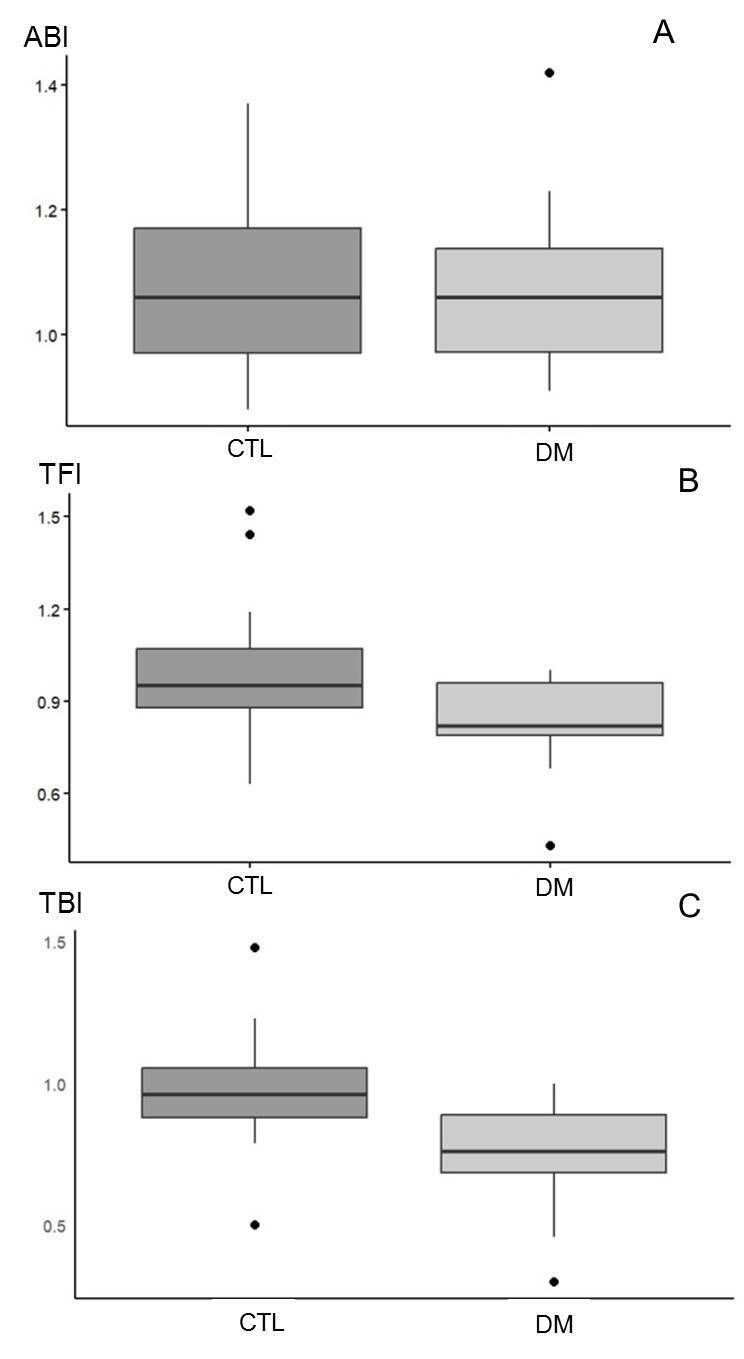
Comparison of the different indices studied in controls (CTL) (n=15) and patients with diabetes (DM) (n=15) (group 2). The central line in the boxplot represents the median and the single points on the diagram show the outliers. (A) ABI = Ankle-brachial index (p=0.92); (B) TFI = Toe-finger index (p=0.03); (C) TBI = Toe-brachial index (p=0.005) (in the *t* test for independent samples; p value <0.05 is considered significant.

Figure 5 shows the comparison of the different indices calculated for the patients with diabetes (DM) and controls (CTL) (group 2). The toe-finger index (Figure 5B) and the toe-brachial index (Figure 5C) were significantly different between CTL and DM groups (p=0.03 and 0.005). No difference in ankle-brachial index (Figure 5A) was observed between the CTL and DM groups (p=0.92). Table 2 shows the characteristics of the participants in the comparative cross-sectional study and Table 3 shows the raw data used for comparison of the different indices studied.

**Table 2:** Characteristics of the participants in the comparative cross-sectional study.

Variable	CTL [mean (SD)/frequency(%)]	DM [mean (SD)/frequency(%)]
Male	7	11
Female	8	4
Age	49.6 (22.4)	62.6 (6.6)
Current smoker	26.6%	13.3%
Hypertension	26.6%	73.3%

CTL = controls; DM = with diabetes.

**Table 3:** Different indices studied in controls (CTL) and in patients with diabetes (DM) (group 2).

ABI - CTL	TFI - CTL	TBI - CTL	ABI - DM	TFI - DM	TBI - DM
1.05	0.92	0.79	0.91	0.99	0.78
1.06	1.07	0.87	0.97	0.68	0.46
1.13	0.98	0.92		0.43	0.30
1.07	1.07	0.96	1.05	0.96	0.66
1.11	1.03	1.23	1.13	0.98	0.89
1.21	1.19	1.48	0.98	0.95	0.72
0.88	1.44	0.96	1.01	0.81	0.59
1.01	0.88	0.89	1.07	0.82	0.76
0.93	0.81	0.96	1.11	1.00	0.76
0.92	0.81	0.81	1.15	0.80	0.71
1.3	1.52	1.02	1.42	0.78	0.89
1.37	0.95	1.09	0.92	0.96	0.94
1.00	0.63	0.50	1.14	0.80	1.00
1.29	0.89	0.89	1.23	0.70	0.90
0.94	0.88	1.13	0.97	0.89	0.78

ABI = Ankle-brachial index; TFI = Toe-finger index; TBI = Toe-brachial index.

## DISCUSSION

Automatic blood pressure measuring devices using oscillometry and optical sensors usually use electric air pumps and solenoid valves to fill (air pump) and deflate (solenoid valve) the cuff. In the device presented herein, a pneumatic air pump was used to control both inflation and deflation of the cuff. This pneumatic motor/pump was an interesting choice during the device manufacturing process, which allowed good control during the cuff deflation phase. Programming embedded in the microcontroller can be modified to adjust different parameters if necessary.

Research has already been carried out on the validity of using oscillometric blood pressure measuring devices to measure the ankle-brachial index. In an integrative review on the subject, it was concluded that devices that use oscillometry to determine ABI did not demonstrate good correlation with measurements performed with a vascular Doppler ultrasound machine and an analog sphygmomanometer device.^13^ In more recent research, the use of oscillometric blood pressure measuring devices for ABI measurement was encouraged, showing good results. However, oscillometric errors occurred more frequently in limbs with peripheral artery disease and legs with incompressible ankle arteries (ABI >1.4) were excluded from the analysis.^14^ Since the oscillometric measurement is calculated by detecting changes in pressure oscillations due to the movement of the arterial wall under the cuff, arterial calcification can make these measurements more difficult.^15^ Current research has already evaluated the possibility of measuring finger pressure by an oscillometric finger-pressing method via smartphones with good results.^16,17^ Regarding the oscillometric measurement of toe pressure, no studies using this technique were found. Most studies use manual blood pressure measurements with a sphygmomanometer and an optical sensor. It is also important to remember that arteries in the foot and toes may have significant calcification, especially in patients at higher risk, such as those with diabetes.^18^ This would theoretically make it even more difficult to measure toe pressure using oscillometry.

In a qualitative evaluation of the prototype under test, it has demonstrated usefulness for assessment of arterial pressure in patients without palpable dorsalis pedis or posterior tibial pulses and with pulses not audible with sonar, that is, patients suspected of having peripheral arterial disease.

We chose to combine two types of research in the same article to indicate possible future research paths for a device that can be used both in specialized vascular surgery centers and in primary health care centers. Both the validation of the device and the proposed clinical research model need to be continued. The toe-finger index, for example, may be evaluated in future research since it proved an interesting option for screening peripheral arterial disease in patients with diabetes in primary health centers.^19^

The correlation of toe pressure between the model device (SysToe) and our prototype was good, showing that in cases of low blood pressure in the extremities (cases in which the device is most useful), the prototype is reliable. In the future, it would be important to increase the number of participants for a more robust correlation. The number of blood pressure measurements taken from the same individual with each device (SysToe and prototype) should also be increased according to the recommendations of the ESH-IP document.^11^ For a more in-depth evaluation of the prototype, the ideal scenario would be to correlate the blood pressures obtained in the hallux with degrees of ischemia in the lower limbs confirmed by imaging methods with arterial US-Doppler or arteriographies.

From a clinical point of view, it was interesting to note that the toe-brachial index was lower in patients with diabetes than in control participants, but the ankle-brachial index was similar in these two groups. This could show the greater sensitivity of this index in mild and moderate vascular disorders. However, the number of participants in this part of the study was too small for such an assessment. Patients with diabetes do not necessarily have PAD. It is possible that the incidence of PAD in the 2 subgroups (controls vs. patients with diabetes) was not statistically different. TFI was also lower in patients with diabetes, in agreement with the TBI findings. Jaffer^19^ compared ABI and photoplethysmography toe-finger index (TFI) measurements for diagnosis of peripheral arterial obstructive disease, finding greater diagnostic sensitivity using TFI in the case of diabetic patients and those with chronic kidney disease. This study, however, also had few participants (n=46), and a larger sample would be necessary to strengthen the findings. For example, analysis of some outliers in our clinical study showed that one participant in the control group with significant smoking showed a discrepantly lower TBI (Figure 5C). A group of heavy smokers or former heavy smokers who stopped smoking less than 10 years previously could be stratified in future research.

Tullos et al.^20^ found an increased risk of abdominal aortic calcification and coronary artery calcification with ABI < 0.9 and even with ABI < 0.99. Combining measurements (ABI, TBI and toe pressure) has proven to be a good option for estimating health risks and sites of arterial obstruction with more precision (without the aid of invasive methods).^21,22^ Measurement of the toe arm index (or TBI) was useful for assessing the chance of postoperative wound healing in obstructive arterial disease, when a higher postoperative TBI was associated with higher odds of wound healing without need for amputation.^23^ Toe pressure in isolation has already been shown to be an important parameter in the prognosis of foot wound healing in diabetic patients.^24,25^

The limitations of the validation study and the comparative clinical study are their small sample sizes. The lack of an accessible diagnostic gold standard for accessing blood pressure in extremities such as fingers and toes made us choose a validated device already on the market as a diagnostic option for comparison. In view of the limitations mentioned, with the growing body of evidence showing the usefulness of measuring toe pressure, TBI, and TFI, the existence of more accessible devices for this purpose is important.

## CONCLUSION

The new device has been shown to be reliable in this initial validation and may therefore have potential for large-scale clinical use. The findings show the feasibility of the device for measuring finger and toe pressures, and possibly greater sensitivity for detecting early-stage arterial disease with this type of measurement.

## References

[1] Eid MA, Mehta K, Barnes JA (2023). The global burden of peripheral artery disease. J Vasc Surg.

[2] Makdisse M, Pereira AC, Brasil DP (2008). Prevalence and risk factors associated with peripheral arterial disease in the Hearts of Brazil Project. Arq Bras Cardiol.

[3] Park SC, Choi CY, Ha YI, Yang HE (2012). Utility of toe-brachial index for diagnosis of peripheral artery disease. Arch Plast Surg.

[4] Tay WL, Lo ZJ, Hong Q, Yong E, Chandrasekar S, Tan GWL (2019). Toe pressure in predicting diabetic foot ulcer healing: a systematic review and meta-analysis. Ann Vasc Surg.

[5] Blaise S, Boulon C, Mangin M (2022). Finger Systolic blood pressure index measurement: a useful tool for the evaluation of arterial disease in patients with systemic sclerosis. Arthritis Care Res.

[6] Vaes RH, Tordoir JH, Scheltinga MR (2013). Blood flow dynamics in patients with hemodialysis access-induced hand ischemia. J Vasc Surg.

[7] Machaczka O, Homza M, Macounová P, Kovalová M, Janoutová J, Janout V (2021). Assessment of toe brachial index validity in diabetic patients: interim results. Vnitr Lek.

[8] Ababneh M, Al Ayed MY, Robert AA, Al Dawish MA (2020). Clinical utility of the ankle-brachial index and toe brachial index in patients with diabetic foot ulcers. Curr Diabetes Rev.

[9] Pérez-Martin A, Meyer G, Demattei C (2010). Validation of a fully automatic photoplethysmographic device for toe blood pressure measurement. Eur J Vasc Endovasc Surg.

[10] Bhamidipaty V, Dean A, Yap SL (2015). Second toe systolic pressure measurements are valid substitutes for first toe systolic pressure measurements in diabetic patients: a prospective study. Eur J Vasc Endovasc Surg.

[11] Stergiou GS, Asmar R, Myers M (2018). Improving the accuracy of blood pressure measurement: the influence of the European Society of Hypertension International Protocol (ESH-IP) for the validation of blood pressure measuring devices and future perspectives. J Hypertens.

[12] Bossuyt PM, Reitsma JB, Bruns DE (2015). STARD 2015: an updated list of essential items for reporting diagnostic accuracy studies. BMJ.

[13] Gengo e Silva RC, Melo VFA, Lima MAM (2014). Validity, reliability and accuracy of oscillometric devices, compared with Doppler ultrasound, for determination of the Ankle Brachial Index: an integrative review. J Vasc Bras.

[14] Hageman D, van den Houten MML, Pesser N, Gommans LNM, Scheltinga MRC, Teijink JAW (2021). Diagnostic accuracy of automated oscillometric determination of the ankle-brachial index in peripheral artery disease. J Vasc Surg.

[15] Benmira A, Perez-Martin A, Schuster I (2016). From Korotkoff and Marey to automatic non-invasive oscillometric blood pressure measurement: does easiness come with reliability?. Expert Rev Med Devices.

[16] Chandrasekhar A, Kim CS, Naji M, Natarajan K, Hahn JO, Mukkamala R (2018). Smartphone-based blood pressure monitoring via the oscillometric finger-pressing method. Sci Transl Med.

[17] Freithaler M, Chandrasekhar A, Dhamotharan V, Landry C, Shroff SG, Mukkamala R (2023). Smartphone-based blood pressure monitoring via the oscillometric finger pressing method: analysis of oscillation width variations can improve diastolic pressure computation. IEEE Trans Biomed Eng.

[18] Liu IH, Wu B, Krepkiy V (2022). Pedal arterial calcification score is associated with the risk of major amputation in chronic limb-threatening ischemia. J Vasc Surg.

[19] Jaffer U, Mohammed Aslam N, Standfield N (2009). Comparison of doppler ultrasound, photoplethysmographic, and pulse-oximetric calculated pressure indices to detect peripheral arterial occlusive disease. Vascular Disease Management..

[20] Tullos BW, Sung JH, Lee JE, Criqui MH, Mitchell ME, Taylor HA (2013). Ankle-brachial index (ABI), abdominal aortic calcification (AAC), and coronary artery calcification (CAC): the Jackson heart study. Int J Cardiovasc Imaging.

[21] Koivunen V, Juonala M, Venermo M, Laivuori M, Jalkanen JM, Hakovirta HH (2021). Toe pressure and toe brachial index are predictive of cardiovascular mortality regardless of the most diseased arterial segment in symptomatic lower-extremity artery disease: a retrospective cohort study. PLoS One.

[22] Fendrik K, Biró K, Endrei D (2023). Screening for peripheral artery disease using an automated four-limb blood pressure monitor equipped with toe-brachial index measurement. J Clin Med.

[23] Luong B, Brown CM, Humphries MD, Maximus S, Kwong M (2023). Assessing the utility of toe arm index and toe pressure in predicting wound healing in patients undergoing vascular intervention. Ann Vasc Surg.

[24] Stone PA, Glomski A, Thompson SN, Adams E (2018). Toe pressures are superior to duplex parameters in predicting wound healing following toe and foot amputations. Ann Vasc Surg.

[25] Zubair M, Al Amri M, Ahmad J (2019). A retrospective study of ABI and TBI during the healing of ulcer among diabetic patients. Diabetes Metab Syndr.

